# Study of Phase Composition and Mechanical Properties of AM50 Magnesium Alloy with Si Addition

**DOI:** 10.3390/ma19091776

**Published:** 2026-04-27

**Authors:** Katarzyna N. Braszczyńska-Malik, Michał Opydo, Jerzy Gęga

**Affiliations:** Department of Materials Engineering, Faculty of Production Engineering and Materials Technology, Czestochowa University of Technology, 19 Armii Krajowej Ave., 42-200 Czestochowa, Poland; michal.opydo@pcz.pl (M.O.); jerzy.gega@pcz.pl (J.G.)

**Keywords:** magnesium alloy, Mg_2_Si, microstructure, mechanical properties

## Abstract

In this paper, the effects of 4 wt.% of silicon on the microstructure and mechanical properties of AM50 magnesium alloys fabricated by the casting method are presented. New AM50/Si material and the base AM50 alloy were gravity cast into a metal mould under the same conditions for comparison. Analyses of the alloys’ microstructures were carried out by light microscopy (with differential interface contrast), scanning electron microscopy (with an energy dispersive X-ray spectrometer), as well as X-ray diffraction (XRD). In as-cast conditions, both materials were composed of α-Mg solid solution, α + γ eutectic (where γ is Al_12_Mg_17_), Al_8_Mn_5_ intermetallic phases and discontinuous γ precipitates. The AM50/Si material also consisted of the Mg_2_Si phase. This structural constituent appeared in the form of primary crystals with regular polygonal morphology and an α + Mg_2_Si eutectic in the form of “Chinese script”. In the microstructure of the AM50/Si material, the Mn_3_SiAl_9_ ternary phase was also identified. The detailed analyses presented in this paper revealed that the new ternary Mn_3_SiAl_9_ structural compound caused a reduction in the volume fraction of the Al_8_Mn_5_ phase but did not completely replace it. These two phases formed competitively. The fabricated material exhibited higher tensile and compression strength as well as yield strength in comparison with the AM50 alloy. Additionally, analyses of the fracture surfaces of the AM50/Si material carried out using scanning electron microscopy (SEM) were presented.

## 1. Introduction

Magnesium alloys are a rapidly developing group of materials with specific properties conditioned by the low density of magnesium itself, which is 1.738 g/cm^3^ (at 293 K) [1,2,3]. The main alloying elements for magnesium are aluminum, zinc, rare earth elements, lithium, or silicon. The most common group of magnesium alloys are materials from the Mg-Al system, in which aluminum provides enhanced mechanical and tribological properties while maintaining the low density of the finished parts. Among these alloys, the most commonly used are AZ-series alloys, such as AZ91 (containing 8.5–9.5 wt.% Al, 0.45–0.9 wt.% Zn, and 0.17–0.4 wt.% Mn), and the AM-series alloys, such as AM50 (containing 4.5–5.3 wt.% Al and 0.28–0.5 wt.% Mn) [4]. The main phase in these alloys (in addition to the α-Mg solid solution) is the intermetallic compound Mg_17_Al_12_ type, which in alloys containing aluminum and zinc has the general formula Mg_17_(Al,Zn)_12_. This compound is often referred to as the γ phase and has a high Young’s modulus of 80 GPa. It is responsible for improving the mechanical, tribological, and corrosion resistance properties of magnesium alloys [4,5,6]. Additionally, manganese in Mg-Al-type magnesium alloys is added primarily to reduce possible iron contamination, which is one of the most unfavourable impurities [4,7,8,9,10]. The formation of four different Al-Mn-type compounds should be expected, i.e., Al_8_Mn_5_, Al_11_Mn_4_, Al_4_Mn and Al_6_Mn, according to the phase diagram presented in [4]. Nevertheless, the Al_8_Mn_5_ phase is the most frequently observed intermetallic compound in cast AM or AZ series alloys.

Recently, materials from the Mg-Si system have also been intensively studied due to a number of properties offered by the main structural component of these materials, i.e., the Mg_2_Si phase, such as high melting point (1358 K), low density (1.99 g/cm^3^), a relatively low coefficient of thermal expansion (7.5 × 10^−6^ K^−1^), high hardness (4.5 × 10^9^ Pa), and a relatively high Young’s modulus (120 GPa) [4,11,12,13,14,15,16,17,18,19,20,21,22,23,24]. According to the Mg-Si phase diagram described in [25], silicon is practically insoluble in magnesium. The maximal solid-state solubility of silicon in magnesium is equal to only 0.003 wt.% at the temperature of eutectic transformation (911.9 K). It should also be noted that eutectic transformation proceeds at 1.48 wt.% silicon, and materials from the Mg–Si system are divided into hypoeutectic, eutectic, and hypereutectic. In the case of hypereutectic materials, the following phase development path can be observed during solidification under equilibrium conditions [11,22,23]:

L → L_1_ + Mg_2_Si_P_ → Mg_2_Si_P_ + (Mg + Mg_2_Si)_E_ (1)

where the index “P” denotes primary particles and “E”—eutectic mixture.

However, under non-equilibrium solidification conditions, the sequence of phase formation can be represented as the following [11,22,23]:

L → L_1_ + Mg_2_Si_P_ → L_2_+ Mg_2_Si_P_ + Mg_P_ → Mg_2_Si_P_ + Mg_P_ + (Mg + Mg_2_Si)_E_ (2)

Analyses of the microstructural investigation of hypo- and hypereutectic materials fabricated from technically pure magnesium and silicon were presented in a previous study [25]. Some studies [14,16,18,19,20,21] have also described the influence of the modification of Mg-Mg_2_Si materials on their microstructure. It should also be noted that due to the properties of the Mg_2_Si phase, materials from the Mg-Si system can also be called in situ composites (especially those with higher levels of silicon), in which the reinforced phase (Mg_2_Si) is formed inside the matrix [23,24,25,26,27,28]. Additionally, it should be noted that the Mg_2_Si compound is also often a component of magnesium matrix composites with SiC particles or aluminosilicate microspheres [24,29,30,31,32,33,34,35,36], or as a reinforcing phase of aluminum matrix composites [37,38,39,40,41,42,43]. On the other hand, materials from the ternary Mg-Al-Si system are another promising group of alloys (or composites). Two main commercial magnesium alloys contain both aluminum and silicon (Si at the 1 wt.% level), i.e., AS21 (containing 2.2 wt.% aluminum) and AS41 (containing 4.2 wt.% aluminum), although alloys with higher levels of aluminum, i.e., AS61 and AS91, have also been developed [3,4].

The development of magnesium alloys requires the search for new material solutions that enable the achievement of complex sets of properties. An approach to the evaluation of these materials is the introduction of additional elements into commercial alloys. This can reduce both technological and material costs. Following this approach, for example alloys based on the AZ and AM series containing rare-earth elements [4,44,45] have been developed. Within this same concept, the authors of [46] produced a material using the AZ91 alloy with 3.34 wt.% Si by spray forming. The effect of silicon addition (up to 1.5%) on the AM series magnesium alloys was also studied in [47,48,49,50], respectively. These materials were produced by the casting method. The papers cited above described the beneficial effect of Si on the mechanical properties (including creep resistance) of magnesium alloys. However, the microstructural analyses in all these works focused on describing the main phases present in these materials, namely the Mg_2_Si and γ phases and the α-Mg solid solution. They did not include analyses of manganese-rich phases.

However, this paper presents a proposal for a material produced on the basis of the AM50 alloy with 4 wt.% Si. The newly developed material was fabricated using a casting method. Particular attention was devoted to microstructural studies and phase analysis of phase components, particularly those containing manganese. The main mechanical properties were also presented. It is well-known that parameters of the fabrication process have a strong influence on the microstructure of cast materials. Therefore, in order to accurately describe the changes in phase composition resulting from the introduction of silicon into the commercial magnesium alloy, as well as its influence on mechanical properties, studies of the AM50 alloy cast under the same conditions are also presented.

## 2. Materials and Methods

A commercial AM50 magnesium alloy in the form of an ingot with a nominal chemical composition of 5 wt.% Al and 0.45 wt.% Mn was used in this study. The actual weight fractions of aluminum and manganese in the obtained casts were confirmed by chemical analysis and were consistent with the attestation. Technically pure silicon in powder form (with irregular morphology and fraction below 1 mm) was introduced into mechanically mixed molten magnesium in order to fabricate an experimental material (referred to as AM50/Mg_2_Si in this work) with 4 wt% of Si. A steel crucible with a capacity of about 1.5 kg of molten magnesium and an argon protective atmosphere were used in the presented casting method. The molten alloy was mechanically mixed, and detailed process parameters were selected experimentally. The fabricated AM50/Si material was gravity cast into a steel mould. An ingot of the pure AM50 magnesium alloy was also remelted and cast under the same conditions for comparison.

For microstructural investigations, samples were cut form the central pats of the fabricated casts and prepared using standard metallographic procedures. To reveal the microstructure, the samples were etched in a 0.5% HNO_3_ solution in C_2_H_5_OH for about 60 s. Microstructural observations were performed using an Olympus GX51 light microscope (Olympus, Tokyo, Japan) with differential interface contrast (LM + DIC). Detailed observations and analyses were made using a JEOL JSM-6610LV scanning electron microscope (JEOL Ltd., Tokyo, Japan) equipped with an energy-dispersive X-ray spectrometer (Oxford Instrument, Abingdon, UK). The phase composition of the presented materials was also confirmed by analysis using X-ray diffraction (XRD). A Brucker D8 Advance diffractometer (Brucker Corporation, Billerica, MA, USA) with Cu_Kα_ X-ray radiation was used in this study.

For the investigated material, the ultimate tensile strength (UTS) and yield strength (TYS) and also the compression strength (CS) and yield strength under compression (YS) were determined. These tests were performed on a Zwick/Roell Z100 machine (Zwick Roell Group, Ulm, Germany). UTS and TYS were tested on standard rod-like samples with a diameter of 8 mm. In the uniaxial compression test, specimens with a diameter of 8 mm and length of 12 mm were used. Both uniaxial tensile and compression tests were carried out with a strain rate of 0.01 mm/s and at room temperature. For comparison, the same mechanical tests were performed on the AM50 magnesium alloy. Three specimens were tested for material. Furthermore, a JEOL JSM-6610LV scanning electron microscope (SEM) (JEOL Ltd., Tokyo, Japan) were used for analysis the fracture surfaces of the AM50/Si material after uniaxial tensile testing.

## 3. Results and Discussion

**Figure 1:**
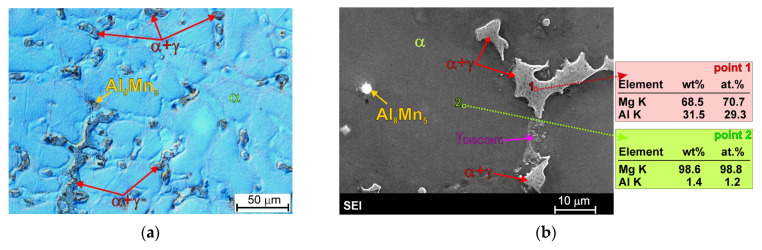
Microstructure images of AM50 alloy (**a**), light microscopy with differential interface contrast; (**b**), scanning electron microscopy with EDX results obtained from designated points).

Figure 1 presents representative micrographs of the microstructure of the as-cast AM50 magnesium alloy. The relatively high tendency of aluminum to segregate in magnesium causes the formation, during non-equilibrium solidification, of large primary dendrites of the α-Mg solid solution (depleted in aluminum—marked as point 2 in Figure 1b). For this reason, at the final stage of solidification, the γ + α eutectic is formed even though the AM50 is a typical hypoeutectic alloy (in which, according to the equilibrium system, eutectic transformation does not occur). This eutectic (marked as point 1 in Figure 1b) has a typical semi-divorced form. Furthermore, detailed SEM observations revealed a relatively small volume fraction of fine (in the initial growth state), secondary, discontinuous γ phase precipitates (described as γ_DISCONT._ in Figure 1b) in the alloy microstructure. Although discontinuous γ phase precipitation is characteristic of T6 heat-treated alloys, such precipitates can also be observed in the structures of cast alloys. These precipitates are also formed in Mg-Al-type cast alloys during cooling of the castings. These are located mainly in interdendritic regions enriched in aluminum due to segregation during solidification.

In addition to the two structural components mentioned above, the microstructure of the commercial AM50 alloy also contains precipitates of a manganese-rich phase, resulting from the presence of this element in its chemical composition. This phase exhibits a blockly morphology (close to round) and, in magnesium casting alloys, most often takes the final form of the Al_8_Mn_5_ intermetallic phase. This structural constituent is located primarily within the α-Mg solid-solution dendrites.

**Figure 2:**
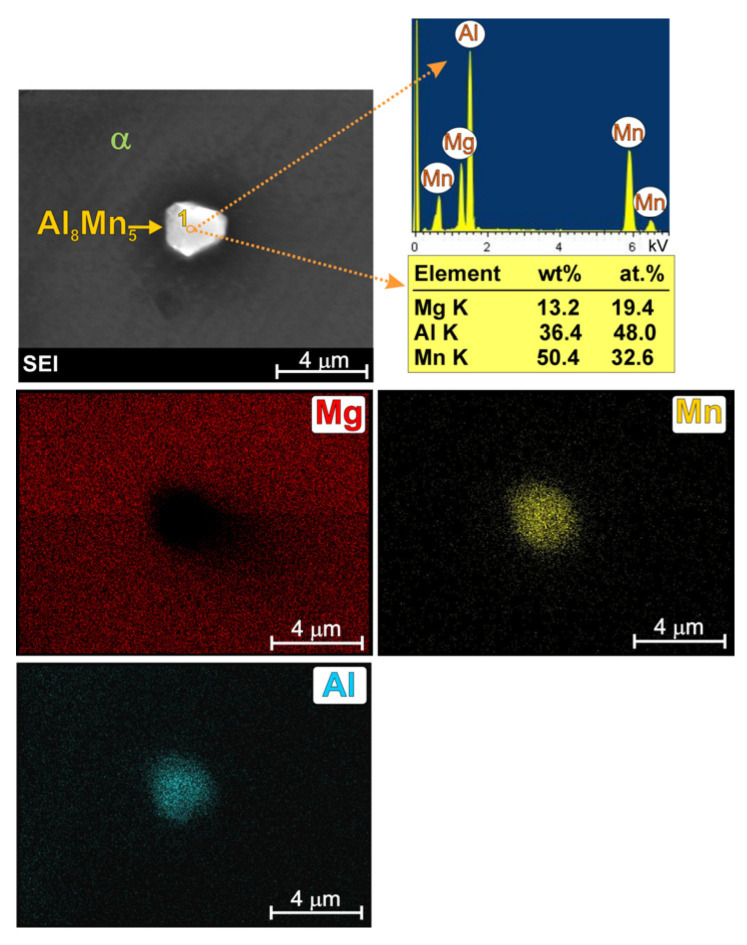
SEM image of Al_8_Mn_5_ phase in AM50 magnesium alloy with EDX results obtained from observed area and designated point.

In the literature on magnesium alloys, the effect of manganese precipitates on the microstructure refinement of these materials is also discussed [4,7,8,9,10]. Some studies [7,9] have concluded that the Al_8_Mn_5_ phase provides favourable nucleation sites for the α-Mg solid solution, thereby contributing to the refinement of the alloy’s primary structure. Other studies [9,10], however, have demonstrated the opposite effect of manganese. On the other hand, according to the thermodynamic calculations described in [4] for the AM50 alloy, the Al_8_Mn_5_ phase is the first to form from the liquid solid solution (the beginning of solidification at 930 K). A detailed image of this phase, together with a point analysis of the chemical composition and surface element distribution is presented in Figure 2.

**Figure 3:**
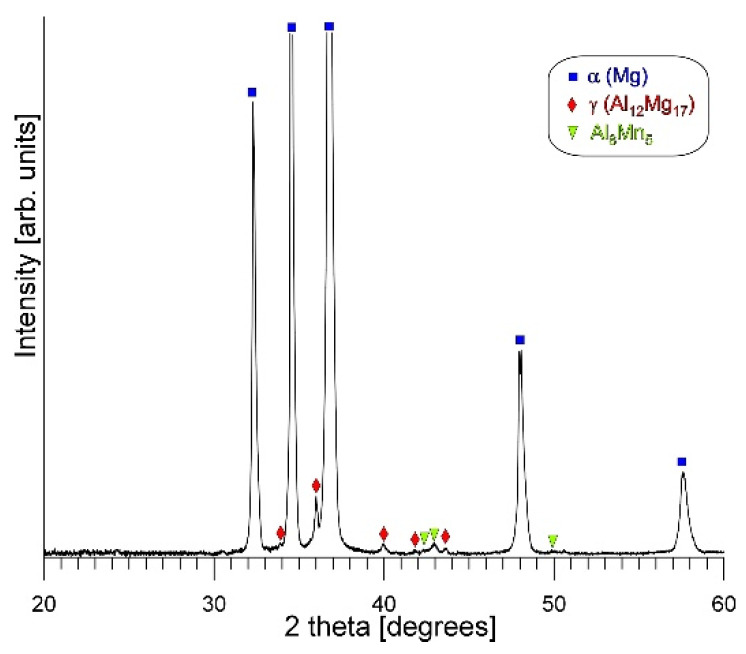
X-ray diffraction pattern of AM50 magnesium alloy.

It should be noted that the penetration depth and width of the electron beam during SEM + EDX analysis are rather high in light magnesium alloys. For this reason, in the results obtained from very fine analyzed participate, the presence of magnesium was also revealed in the point analysis (originating from the background). Nevertheless, XRD analysis was performed to confirm the phase composition of the investigated alloy. Figure 3 presents the X-ray diffraction patterns obtained for the cast AM50 alloy.

**Figure 4:**
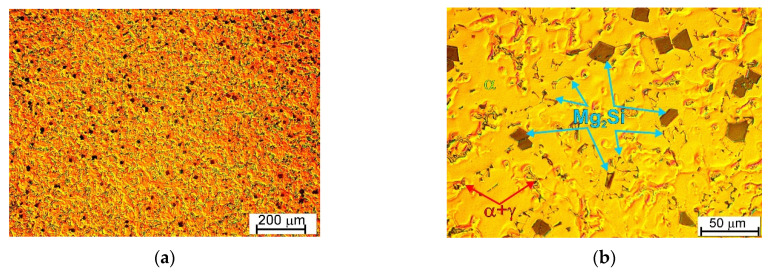
Microstructure images of AM50/Si material ((**a**,**b**), micrographs taken at different magnifications of various areas of etched surface, light microscopy with differential interface contrast).

**Figure 5:**
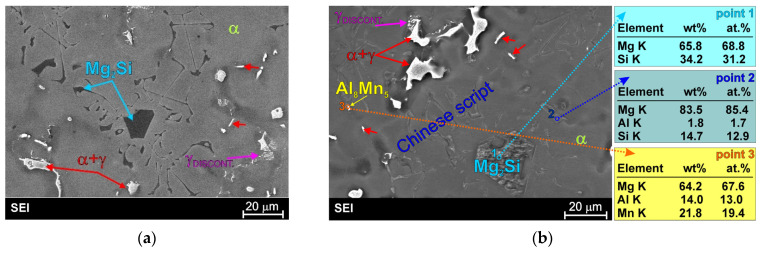
Microstructure images of AM50/Si material ((**a**,**b**), micrographs taken from different areas; scanning electron microscopy with EDX results obtained from designated points).

Figure 4 and Figure 5 show representative micrographs of the microstructure of the as-cast AM50 magnesium alloy with 4 wt.% Si, which was successfully fabricated by the casting method. The microstructure of AM50/Si materials was characterized by a homogeneous distribution of structural components and also by a dendritic morphology of the α-Mg solid solution. The microstructure of the produced material consist of primary Mg_2_Si in the characteristic form of blocky, regular polygonal morphologies typical for faceted crystals. This shape can be described as an octahedron, hexahedron, or tetrakaidecahedron. Such morphologies were also observed in a previous study on Mg-Mg_2_Si materials fabricated on the basis of technically pure magnesium and cast under analogous conditions [25]. It should also be noted that different primary Mg_2_Si structural compound shapes have been previously observed [11,12,13,14,15,16,17,18,19,20,21]; however, as concluded in [25], this depends on the chemical composition of the material (and the presence of further elements or impurities), the cooling rate, and the degree of supercooling during solidification.

The next structural constituent in the investigated material is the α + Mg_2_Si eutectic, which exhibits an irregular morphology, typical of a faceted–nonfaceted eutectic mixture. However, it should be emphasized that, in comparison with the Mg-Mg_2_Si-type materials (cast on the basis of technically pure magnesium) [25], the eutectic formed in the investigated material based on the AM50 alloy was characterized by a different grained structure, which could be described as “Chinese script”. In the microstructure of the analyzed material, the same structural constituents as in the AM50 base alloy were also observed, i.e., the α-Mg solid solution depleted in aluminum, the γ + α semi-divorced eutectic, discontinuous γ phase precipitates, and a reduced volume fraction of fine Al_8_Mn_5_ intermetallic compound.

**Figure 6:**
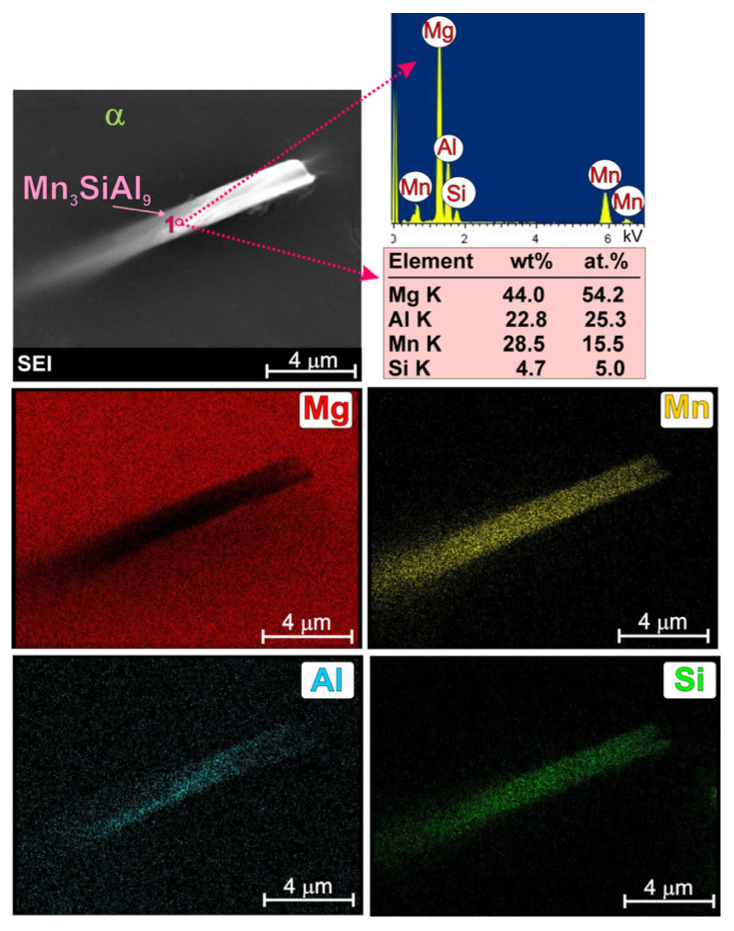
SEM image of Mn_3_SiAl_9_ phase in AM50/Si material with EDX results obtained from observed area and designated point.

**Figure 7:**
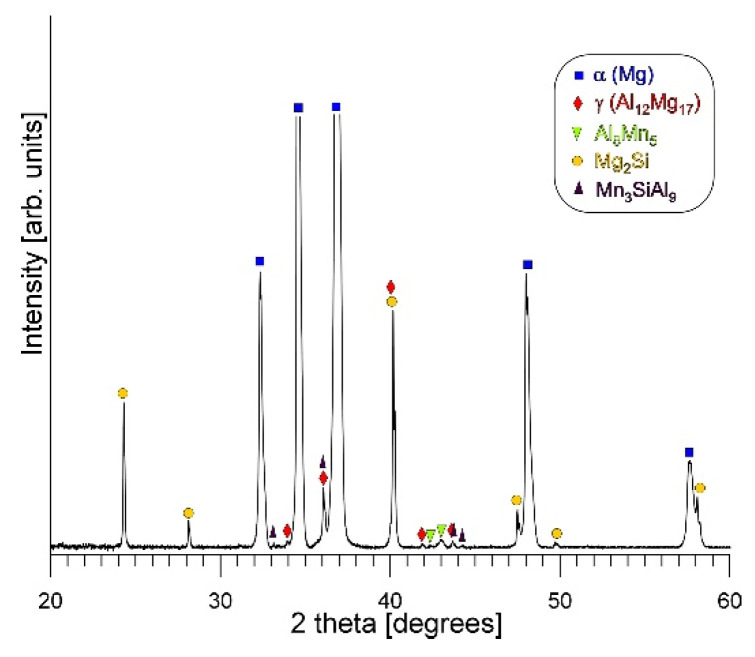
X-ray diffraction pattern of AM50/Si material.

Detailed observation of the microstructure of the fabricated AM50-Si material using a scanning electron microscope also revealed the presence of fine, needle-like precipitates (marked with red arrows in Figure 5). Separate, detailed investigations of this phase, together with a point analysis of the chemical composition and surface element distribution are shown in Figure 6. The results obtained using an energy dispersive X-ray spectrometer clearly reveal the simultaneous presence of aluminum, manganese, and silicon in the observed needle-like precipitates. Although the presented results of SEM + EDS investigations unequivocally indicate the presence of the above elements in the chemical composition of the observed precipitates, they do not allow assigning a specific formula to this compound, mainly due to the presence of an atomic fraction of magnesium in the phase composition (from the background). The phase composition of the investigated material was determined on the basis of X-ray phase analysis. Figure 7 shows the X-ray diffraction patterns obtained for the cast AM50 alloy with Si. The result of these analyses confirmed the presence of the phases described above, i.e., α-Mg, γ, Al_8_Mn_5_, Mg_2_Si. The X-ray analyses also revealed a significant presence of the ternary Mn_3_SiAl_9_ compound.

In previous studies of materials prepared from commercial magnesium alloys (AZ91, AM50 or AM60) with Si addition [46,47,48,49,50], the presence of manganese-rich structural compounds was not analyzed, despite their presence in the base alloys. The presence of neither a binary nor a ternary phase containing manganese was considered. As the results presented in this article demonstrate, manganese-rich phases also occur as structural separated components in these materials. It should also be noted that although the presence of the ternary phase Mn_3_SiAl_9_ in magnesium materials has not been previously reported, it has been described in the Al-Si-Mn system [51,52,53]. Additionally, this compound with needle-like morphology was also observed in commercial aluminum alloys, such as the 6082 alloy [54,55]. It should also be noted that in the investigated material, the new ternary Mn_3_SiAl_9_ phase did not completely replace Al_8_Mn_5_.

**Figure 8:**
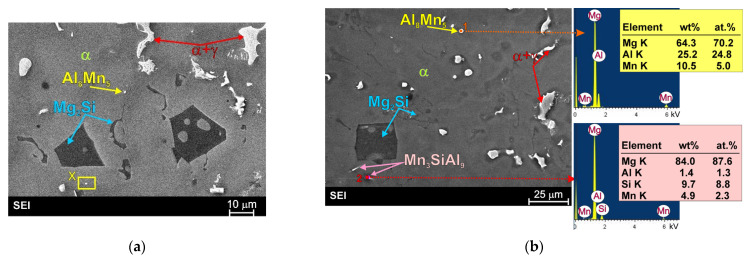
SEM images present phases in AM50/Si material with EDX results obtained from designated point ((**a**,**b**), micrographs taken at different magnification of various areas of etched surface).

**Figure 9:**
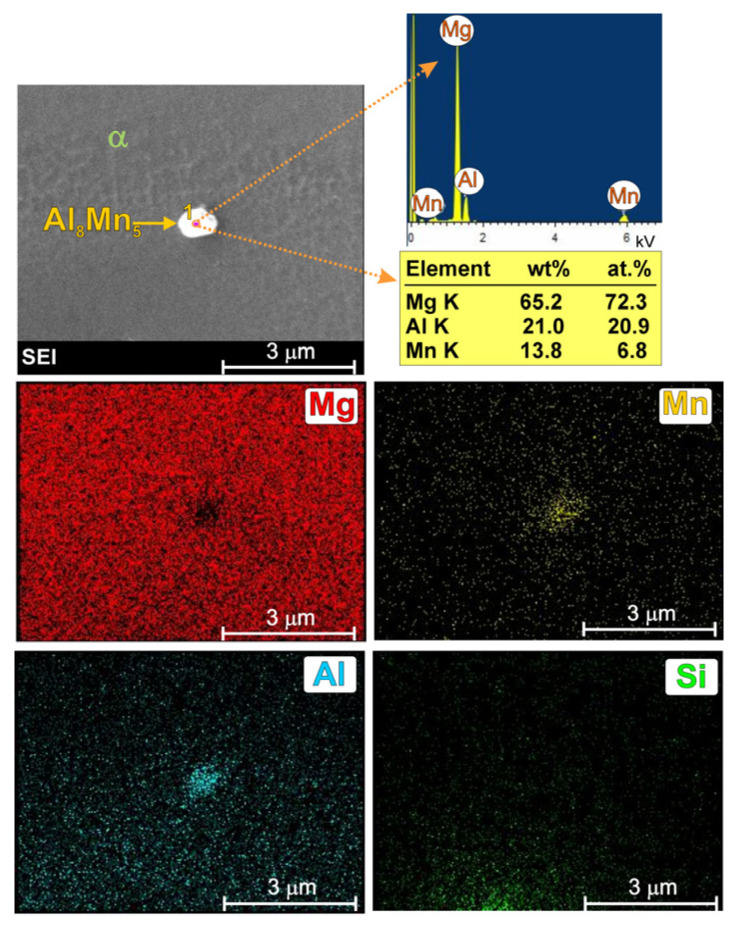
SEM image of Al_8_Mn_5_ phase in AM50/Si material with EDX results obtained from observed area and designated point (higher magnification of area marked as X on Figure 8a).

Although the Al_8_Mn_5_ compound was close to the XRD detection limit, careful analyses using both X-ray and SEM + EDX techniques confirmed the presence of both Al_8_Mn_5_ and Mn_3_SiAl_9_ phases in the investigated AM50 magnesium alloy with silicon. Figure 8 represents both the Al_8_Mn_5_ phase (marked as point 1 in Figure 8b—rich in aluminum and manganese) and the Mn_3_SiAl_9_ phase (marked as point 2 in Figure 8b—rich in aluminum, manganese and silicon). Separate, detailed investigations of the Al_8_Mn_5_ phase in AM50/Si material, together with a point analysis of the chemical composition and surface element distribution are also shown in Figure 9.

It should therefore be assumed that both phases formed competitively during solidification, depending on local fluctuations in the chemical composition of the alloy. Due to the limited weight fraction of manganese in the investigated material, the formation of the precipitated Mn_3_SiAl_9_ compound caused a reduction in the volume fraction of the Al_8_Mn_5_ phase but did not completely replace it. This contrasts with the results obtained for AM- or AZ-series commercial alloys after the addition of rare-earth (RE) elements [44,45]. The introduction of RE elements (in the form of cerium-rich mischmetal) into AM50 or AZ91 alloys caused the formation of a new ternary Al10RE2Mn7 intermetallic compound but instead of the Al_8_Mn_5_ phase.

**Figure 10:**
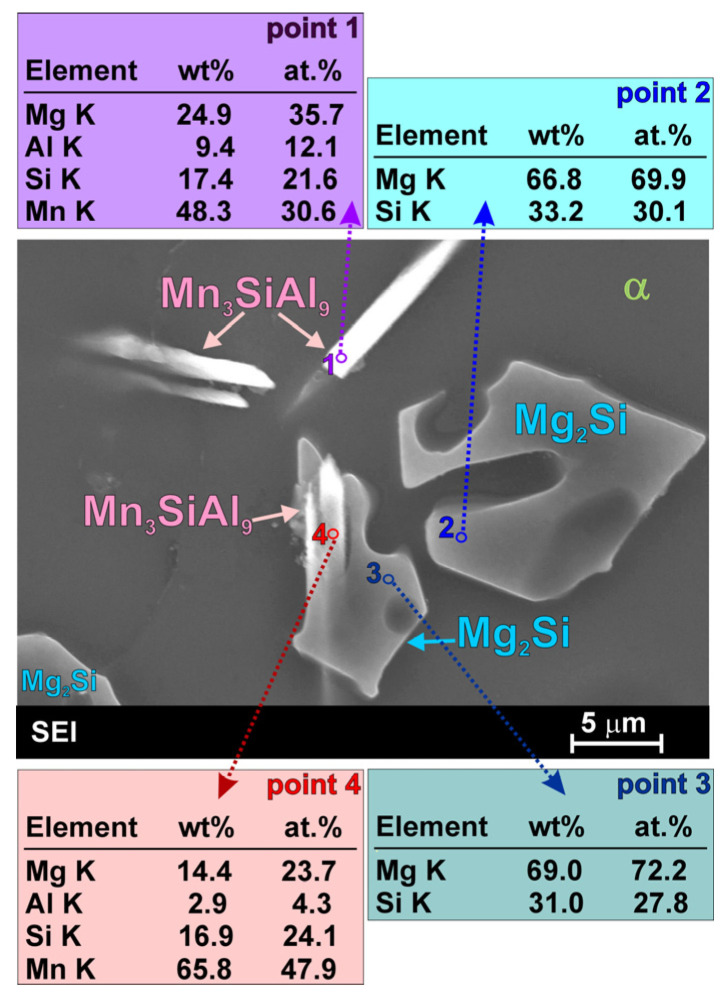
SEM image of some phases in AM50/Si material with EDX results obtained from designated points.

Particles of the ternary Mn_3_SiAl_9_ compound with needle-like morphology were frequently observed within the α-Mg solid solution, as shown in Figure 5 and Figure 8. It should be noted, however, that this phase was also observed in some cases within the primary Mg_2_Si structural compound. Figure 10 presents an SEM image in which the Mn_3_SiAl_9_ phase is visible inside both the α-Mg solid solution (point 1) and the Mg_2_Si phase (point 4). Such positioning of this phase may indicate its heterogeneous influence on the nucleation of individual structural constituents.

It is well-known that a necessary condition for heterogeneous nucleation is the formation of a coherent or at least semi-coherent interface with a small lattice mismatch (less than 0.1) along the interface between the nucleus and the substrate. Although both magnesium and the Mn_3_SiAl_9_ phase have the same hexagonal structure (*P63*/*mmc space group*), theoretical calculation mismatches in the main direction of those phases are much greater than 0.1. On the other hand, according to typical crystallographic calculations, Mg_2_Si (with an *Fm-3m cubic crystal structure*) and Mn_3_SiAl_9_ exhibit a lattice misfit of up to 0.1. The smallest interatomic spacing mismatch occurs along the main directions of the basal planes but is still larger than 0.1 (equal to 0.155). These values indicate a random distribution of the phase in the alloy volume, resulting from local crystallization conditions and fluctuations in chemical composition during solidification.

**Figure 11:**
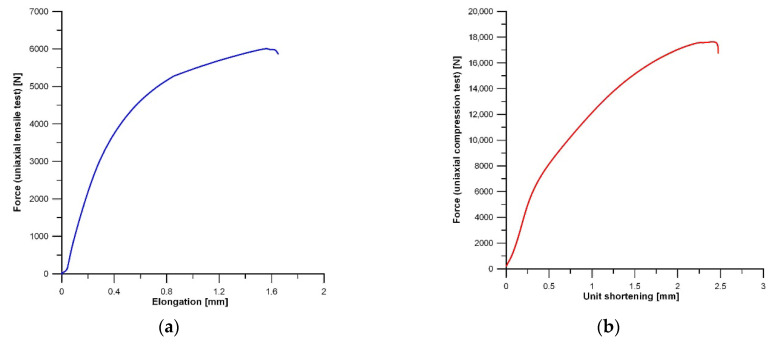
Representative tension (**a**) and compression (**b**) curves for AM50/Si material.

**Figure 12:**
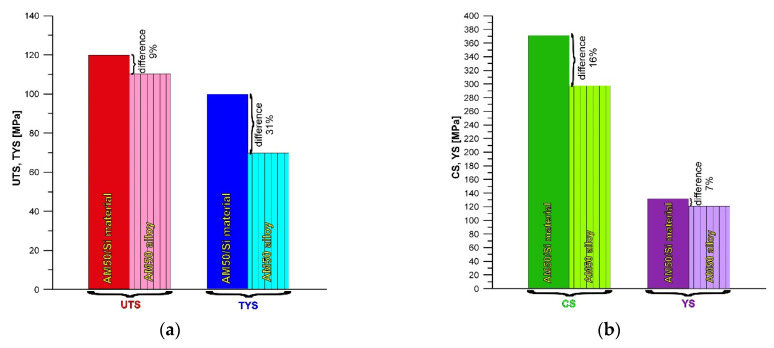
Ultimate tensile strength (UTS), yield strength (TYS) (**a**), compression strength (CS) and yield strength under compression (YS) (**b**) of AM50/Si material compared with AM50 alloy.

The representative tension and compression curves recorded for the AM50/Si material are shown in Figure 11. Figure 12 presents the values obtained in the uniaxial tensile and compression tests for the AM50/Si material and, for comparison, for the AM50 alloy cast under the same conditions. In both uniaxial tensile and uniaxial compression tests at room temperature the fabricated material with the Si addition exhibited higher mechanical properties than the AM50 alloy. For the investigated AM50/Si material, the ultimate tensile strength (UTS) and the yield strength (TYS) were 120 and 100 MPa, respectively. The compression strength (CS) and yield strength under compression (YS) were 351 and 130 MPa. The largest difference in properties between these materials was observed in the yield strength (TYS), which was equal to 31%. On the other hand, the AM50/Si material had an ultimate tensile strength (UTS) only 9% higher than the AM50 alloy. The results obtained are consistent with those reported by Young et al. [49], which demonstrates a beneficial effect of silicon addition on the mechanical properties (in the uniaxial tensile test) of the AM60 alloy, although this effect was approximately 11% at most. The AM50/Si material also exhibited the compression strength (CS) and yield strength under compression (YS) 16% and 7% higher, respectively, than for the AM50 alloy.

**Figure 13:**
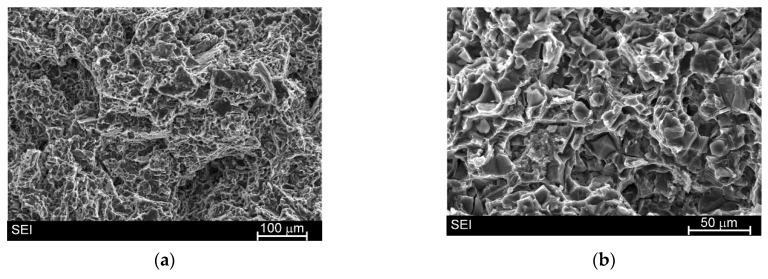
SEM images of fracture surface of AM50/Si material after uniaxial tensile test ((**a**,**b**), micrographs taken at different magnification of various areas of fracture surface).

Figure 13 shows the fracture surface of the AM50/Si material. It is well-known that due to the hexagonal closed packed structure of Mg, magnesium alloys very often exhibit rather brittle through cleavage or quasi-cleavage fracture. The fracture surface of the AM50/Si material was characterized by dimples and river patterns. On this surface were also clearly visible the primary Mg_2_Si phase. These particles were also cracked (and exhibited intrinsic brittleness). The cracking process proceeded through the Mg_2_Si particles with the propagation of secondary cracks proves a strong bond between the Mg_2_Si phase and the magnesium alloy. The same results were also observed for Mg_2_Si particles in Mg/Mg_2_Si composites [23,25]. On the other hand, the manganese-rich phases (described above) were too fine to be identified on the fracture surfaces.

## 4. Conclusions

The influence of silicon on the microstructure characterization of AM50 magnesium alloys was investigated, and the following results were obtained:

1. The new AM50/Si material was successfully fabricated using the casting method.
2. The microstructure of the AM50/Si material consisted of α-Mg solid solution, α + γ eutectic (where γ is Al_12_Mg_17_) and Al_8_Mn_5_ intermetallic phases, the Mn_3_SiAl_9_ ternary compound, and the Mg_2_Si phase.
3. The Mg_2_Si phase appeared in the form of primary crystals with regular polygonal morphology, and the α + Mg_2_Si eutectic in the form of “Chinese script”.
4. The Mn_3_SiAl_9_ ternary structural compound was found for the first time in AM50/Si material, which caused a reduction in the volume fraction of the Al_8_Mn_5_ phase but did not completely replace it.
5. The fabricated AM50/Si material exhibited higher mechanical properties than the AM50 alloy. The cracking process of the investigated material proceeded mainly through the main phases (including the Mg2Si phase).

## Data Availability

The original contributions presented in this study are included in the article. Further inquiries can be directed to the corresponding author.
